# *Brassica rapa* hairy root based expression system leads to the production of highly homogenous and reproducible profiles of recombinant human alpha‐L‐iduronidase

**DOI:** 10.1111/pbi.12994

**Published:** 2018-08-30

**Authors:** Florian Cardon, Roser Pallisse, Muriel Bardor, Aurore Caron, Jessica Vanier, Jean Pierre Ele Ekouna, Patrice Lerouge, Michèle Boitel‐Conti, Marina Guillet

**Affiliations:** ^1^ Root Lines Technology SA Amiens France; ^2^ Laboratoire Glyco‐MEV EA4358 UNIROUEN Normandie Université Rouen France; ^3^ Institut Universitaire de France (I.U.F.) Paris Cedex 05 France; ^4^ Biologie des Plantes et Innovation (BIOPI) Université de Picardie Jules Verne Amiens France

**Keywords:** hairy root, plant based expression system, recombinant, glycoprotein, glycosylation, alpha—L—iduronidase, post‐translational modification

## Abstract

The *Brassica rapa* hairy root based expression platform, a turnip hairy root based expression system, is able to produce human complex glycoproteins such as the alpha—L—iduronidase (IDUA) with an activity similar to the one produced by Chinese Hamster Ovary (CHO) cells. In this article, a particular attention has been paid to the *N*‐ and *O*‐glycosylation that characterize the alpha‐L‐iduronidase produced using this hairy root based system. This analysis showed that the recombinant protein is characterized by highly homogeneous post translational profiles enabling a strong batch to batch reproducibility. Indeed, on each of the 6 *N*‐glycosylation sites of the IDUA, a single *N*‐glycan composed of a core Man_3_GlcNAc_2_ carrying one beta(1,2)‐xylose and one alpha(1,3)‐fucose epitope (M3XFGN2) was identified, highlighting the high homogeneity of the production system. Hydroxylation of proline residues and arabinosylation were identified during *O‐*glycosylation analysis, still with a remarkable reproducibility. This platform is thus positioned as an effective and consistent expression system for the production of human complex therapeutic proteins.

## Introduction

To date, various recombinant protein expression systems have been developed. This can be explained by the constraints that each of these systems impose: inability to produce and/or secrete functional complex proteins (e.g.: bacterial systems), existence of a risk of viral transmission and toxic molecules (e.g.: bacterial systems, mammalian cells), societal rejection (e.g.: GMO plants in fields), or high production costs (e.g.: mammalian cells). Thus, overall, all of the existing production systems present some limitations.

Since more than 25 years, the transgenic plants have appeared as an alternative system for the production of heterologous recombinant therapeutic proteins offering a number of major advantages as compared to the usual production from industrial cell lines among which, in particular, the absence of potential contamination by animal pathogens and the possibility of mass production at low cost. However, the culture of transgenic plants in open fields or in greenhouses is associated with numerous limitations such as social rejection or environmental influence. This has led the scientific community to develop plant alternatives combining the intrinsic advantages of plants and a possible production confinement. In this context, the hairy root expression system appears as an obvious favourable candidate.

Hairy roots emerge from the wounding site of plantlets after the infection by a symbiotic bacterium called *Rhizobium rhizogenes*. In nature, this phenomenon is beneficial for the infected plants as it enables them to extract more soil nutrients and water. For its counterpart, the bacterium builds an environment favourable to its development. These additional not adventitious roots possess the quality to grow indefinitely and without geotropism.

Hairy roots have been widely studied and used for the production of specialized/secondary metabolites of industrial and pharmaceutical interest (Georgiev *et al*., 2007; Giri and Narasu, 2000; Guillon *et al*., 2006a,b; Srivastava and Srivastava, 2007). Since the 1990s, the production of recombinant proteins has been considered as another promising application of hairy root cultures. The first proof of concept was achieved by producing a mouse monoclonal antibody by hairy roots of tobacco plants (Wongsamuth and Doran, 1997). It was shown that this antibody was secreted and accumulated in the culture medium. Other recombinant proteins have been produced and secreted by tobacco hairy roots, including the green fluorescent protein (Medina‐Bolívar and Cramer, 2004), the murine interleukin (Liu *et al*., 2009), the human acetylcholinesterase (Woods *et al*., 2008) or the thaumatin sweetener (Pham *et al*., 2012). In order to improve the hairy‐root based expression system, a plant species from the *Brassicaceae* family was especially selected because it met certain criteria like use of an edible plant to avoid any risk of known toxicity and high heterologous protein secretion capacity by transformed roots (Huet *et al*., 2014). In addition, the hairy roots that emerge from *Brassica rapa* can grow indefinitely, which is not the case for the hairy roots that emerge from *Nicotiana benthamiana* for example (Huet *et al*., 2014). Using this system, complex recombinant proteins can be produced (Ele Ekouna *et al*., 2017). In the present article, the in‐depth characterization of one of such recombinant protein, referred to as ‘rIDUA_RLT’ (for recombinant alpha‐L‐iduronidase_Root Lines Technology), is described.

The alpha‐L‐iduronidase (IDUA) is a complex human glycoprotein which deficiency leads to the development of the mucopolysaccharidosis type I (MPS I), a progressive lysosomal storage disorder. Alpha‐L‐iduronidase (IDUA; EC 3.2.1.76) is a 71 kDa lysosomal enzyme that hydrolyses the terminal alpha‐L‐iduronic acid residues of the glycosaminoglycans, such as dermatan sulphate and heparan sulphate. IDUA is a secreted protein presenting a signal peptide (^1^M‐^23^A) which is released in its final secreted form and six potential *N*‐glycosylation sites as well as hydroxylation. Such protein have already being produced in plants (Acosta *et al*., 2015; He *et al*., 2012, 2013; Pierce *et al*., 2017) and in CHO cells (Aldurazyme^®^, Sanofi, Paris, France; Kakkis *et al*., 1994). This gave the opportunity to compare the characteristics of the rIDUA_RLT protein produced in hairy root clones with its benchmark produced by using a mammalian‐based production system or other production systems.

Here, in order to biochemically characterize the protein produced by this hairy roots based expression platform, we analysed its activity as well as its post‐translational modifications. As most of the biopharmaceuticals are glycosylated proteins (Walsh and Jefferis, 2006) and as it is well‐established that the glycosylation of these recombinant proteins are essential for their biological activity, safety, efficacy and immunogenicity (van Beers and Bardor, 2012; Lingg *et al*., 2012), a particular attention was paid to the analysis of both the *N*‐ and *O*‐glycosylation profiles of the protein produced using the hairy root‐based expression system.

## Results

### Transformed turnip hairy root clones are able to reproducibly secrete an active rIDUA_RLT recombinant protein that can be purified using a customized process

**Figure 1:**
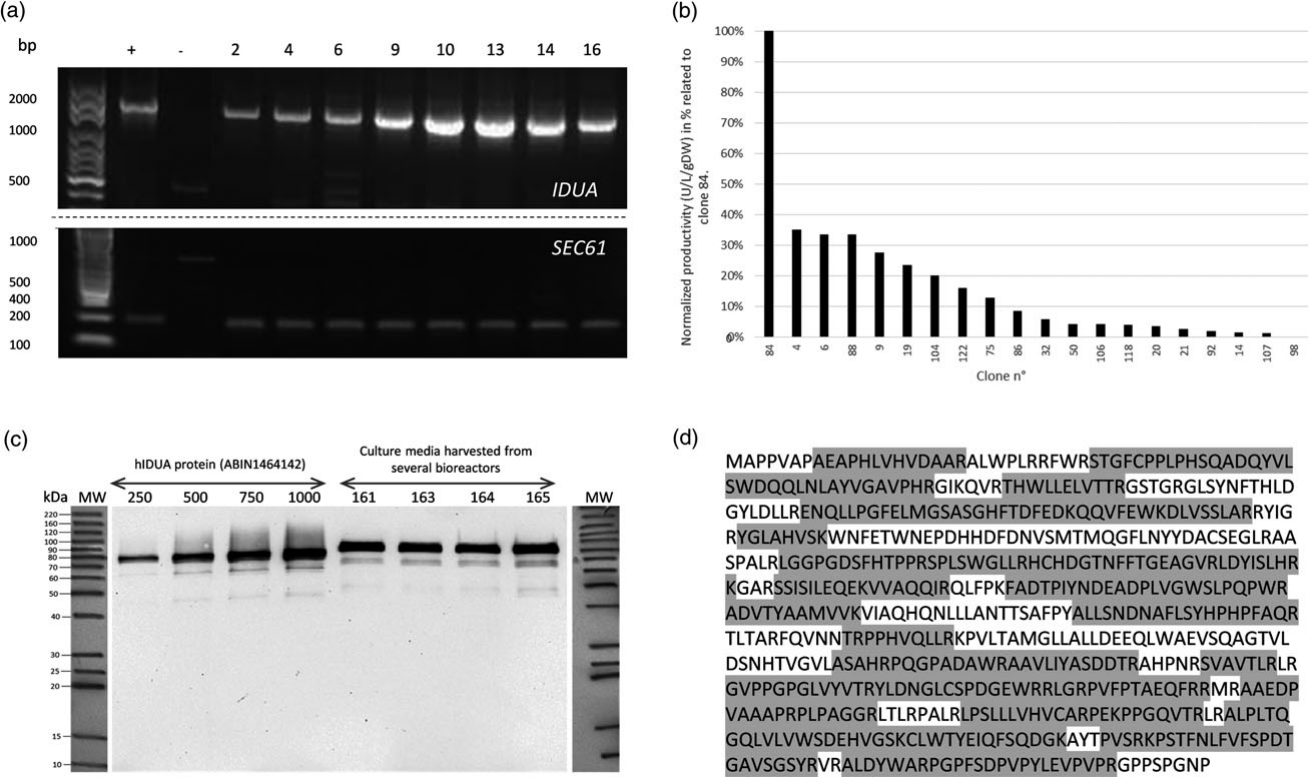
(a) Example of transgene expression detection by RT‐PCR. (Top gel) PCR detection of the IDUA transgene expression from total RNA extracted from different hairy root clones. (+) pRD400 plasmid with IDUA gene was used as positive control, (−) RNA extract of wild type hairy root clone (without any transfection) was used as negative control (Bottom gel) PCR detection of a gene constitutively expressed in Brassica: SEC61 (endoplasmic reticulum membrane protein translocator) (+) RNA extract of wild type hairy root (without any transfection) was used as positive control, (−) pRD400 plasmid with IDUA gene was used as negative control, numbers designating clones, DNA ladder in base pair (bp). (b) Normalized productivity (U/L/g dried weight) in percentage related to the best producing clone, namely clone 84. (c, d) Identification of rIDUA_RLT in crude culture media by (c) Western blot analyses of equivalent amounts of four different batches (161, 163, 164, 165) of the selected hairy root clone 84 in comparison with a commercial standard protein (ABIN1464142, Antibodies‐online) in increasing amount (250, 500, 750, 1000 ng), (d) Mass spectrometry analysis of rIDUA_RLT. The identified peptides are highlighted in grey on the amino acid sequence.

Turnip plantlets were infected with transformed *R. rhizogenes* containing the plasmid pRD400‐SP‐IDUA as described previously (Huet *et al*., 2014). One hundred and fifty‐three hairy root clones emerged from the wounding sites. These clones were individualized and cultured first in solid and then in liquid media. At this stage, 39 hairy root clones were selected for their growth capacity through a phenotypic screen (2 cm‐long roots or higher). Hairy root clones having stably integrated the human IDUA transgene were selected through a PCR analysis on hIDUA transcripts using specific primers for IDUA and the SEC61 gene, which is constitutively expressed in Brassica species [UniProtKB ‐ P0DI74 (S61G1_ARATH)] (Figure 1a). After this selection step, the ability of the selected hairy root clones to produce and secrete an active recombinant α‐L‐iduronidase protein into the media was assessed. As shown in Figure 1b, productivity observed for clone 84 was outstanding as compared to the others. This best producing clone was finally selected for further analyses based on the activity assay screening. The immunodetection analysis of the culture medium from this selected hairy root clone using an anti‐IDUA antibody shows a band at a molecular weight of nearly 80 kDa. According to the fact that the molecular weight of the mature non glycosylated protein is theoretically expected to be 70.77 kDa, this result confirmed the presence of the protein of interest in the culture medium under a glycosylated form (Figure 1c). This analysis was done using the crude culture medium harvested from several bioreactors cultured from the same hairy root clone (clone 84) showing a remarkable batch‐to‐batch reproducibility of the production of rIDUA_RLT protein by the selected clone (Figure 1c). Finally, the identity of the protein of interest was further validated by a proteomic approach combined to MS^2^ from crude culture medium. This analysis displayed a 68.5% peptide recovery as compared to the theoretical sequence of the expressed IDUA (Figure 1d).

**Figure 2:**
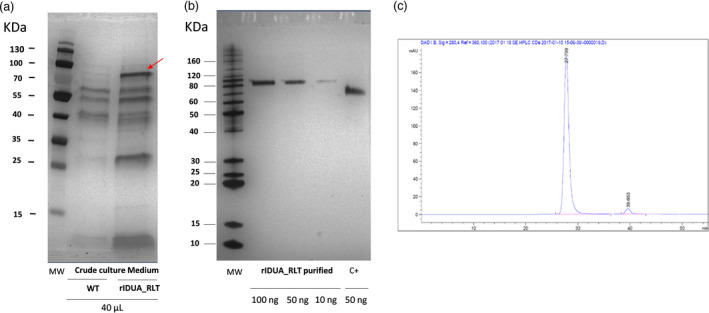
(a) Silver stained SDS‐PAGE gel of crude culture media from a wild type (WT) hairy‐root clone (hairy root clone without any integrated transgene) and the selected hairy root clone 84. Red arrow points out the rIDUA_RLT (b) Silver stained SDS‐PAGE gel of the purified rIDUA_RLT protein (100, 50 and 10 ng of rIDUA_RLT) and 50 ng of positive control C+ (ABIN1464142, Antibodies‐online) (c) SE‐HPLC profile of the purified rIDUA_RLT protein. The purity of the rIDUA_RLT is of 96.2% without any soluble aggregates.

The rIDUA_RLT protein (Figure 2a) was then purified using a two chromatographic step protocol. A protein purity degree of over 96% (Figure 2b) without any soluble aggregate as estimated by SE‐HPLC and RP‐HPLC was obtained (Figure 2c; see Data S1).

### The *N‐*terminal sequence of rIDUA_RLT corresponds to the expected sequence, demonstrating that the signal peptide was efficiently removed

For the recombinant protein to be secreted within the culture medium, a gene sequence encoding for a specific signal peptide already used in (Ele Ekouna *et al*., 2017; Huet *et al*., 2014) was added to the *5′* end of the nucleotide sequence. This signal peptide has to be removed during the secretion step, in the endoplasmic reticulum as normally observed in eukaryotic cells (Dudek *et al*., 2015; Kober *et al*., 2013; Zimmermann *et al*., 2011). To validate the absence of the signal peptide in the protein secreted by the hairy roots based expression platform, a *N*‐terminal sequencing using Edman degradation was performed (data not shown). A *N*‐terminal MAXXV pentapeptide was identified with X being a modified amino acid. Considering that the expected *N*‐terminal pentapeptide is MAPPV, this analysis suggested that cleavage of the signal peptide occurred as expected before the Met 24. This demonstrates the efficiency of the hairy root expression system to recognize and cleave successfully the signal peptide of heterologous proteins.

### rIDUA_RLT protein exhibits enzymatic kinetics that are comparable with the ones displayed by the same enzyme produced in CHO cells

**Figure 3:**
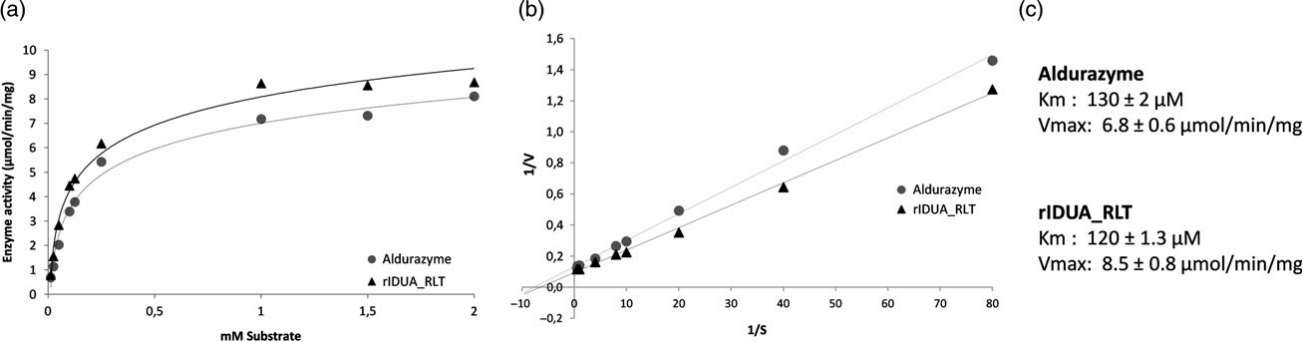
(a) Michaelis‐Menten curves and (b) Lineaweaver‐Burk kinetic assay plots for Aldurazyme and the recombinant IDUA produced in transgenic turnip hairy roots (rIDUA_RLT). (c) Kinetic parameters of *K*_m_ and *V*_max_ values are an average of three independent experiments ± SD.

The kinetic parameters of the purified rIDUA_RLT protein produced were measured. Michaelis‐Menten kinetics were used to characterize the enzymatic properties of the rIDUA_RLT protein in comparison with the commercially available recombinant protein produced in CHO cells (Aldurazyme^®^, Sanofi). rIDUA_RLT displays a Vmax of 8.5 μmol/min/mg, a kcat of 11.8 s^−1^ and a Km of 120 μm, whereas Aldurazyme, analysed in parallel within the same experiment, exhibited a Vmax of 6.8 μmol/min/mg, a kcat of 9.4 s^−1^ and a Km of 130 μm. These results show that rIDUA_RLT and Aldurazyme have comparable enzymatic activity and affinities towards the same substrate (Figure 3). The enzymatic activity being usually related to the protein quality and glycosylation, it was of interest to biochemically characterize the rIDUA_RLT protein and focus especially on its glycosylation profile.

### *N*‐Glycosylation analysis indicates that rIDUA_RLT protein displays a highly homogeneous paucimannosidic profile

**Figure 4:**
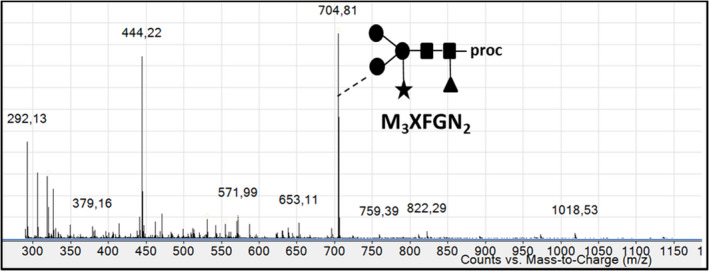
ESI mass spectrum of N‐glycans isolated from rIDUA_RLT and coupled to procainamide. Ion at m/z 704.81 was assigned to the doubled charged [M + 2H]^2+^ M3XFGN2. Other ions correspond to non‐glycan contaminants. M, mannose; X, xylose; F, fucose; GN, N‐acetylglucosamine.

**Figure 5:**
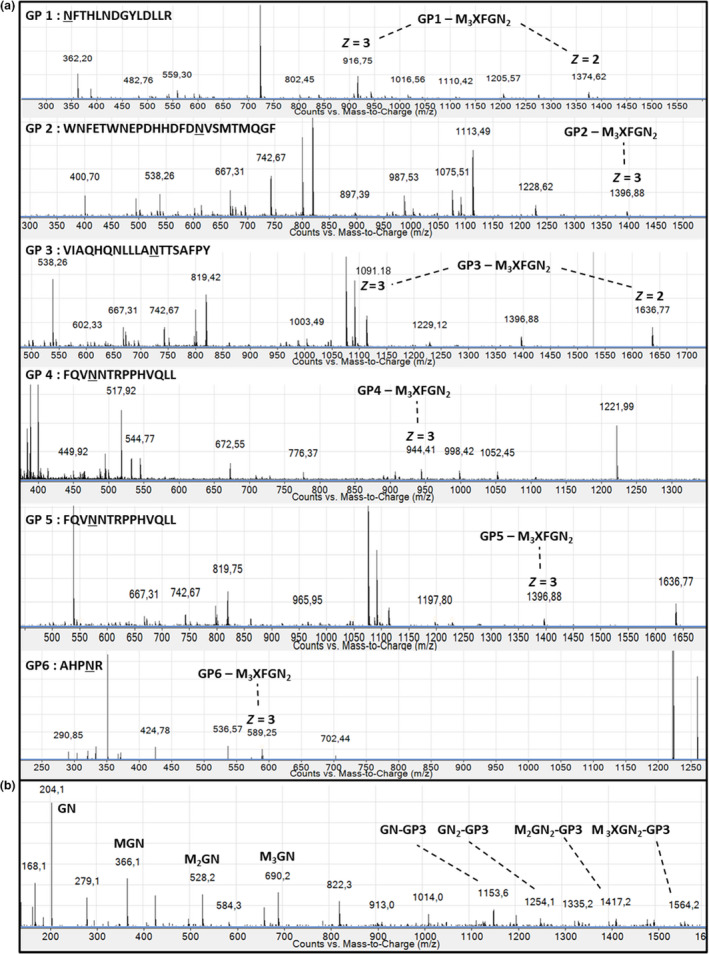
(a) nano ESI mass spectra of LC peaks from digested rIDUA_RLT with MS2 spectra exhibiting N‐glycan reporter fragment ions at m/z 204, 163 and 366. In each MS spectrum, glycopeptide exhibits different charge states (*z* = 2 or 3). In the peptide sequence, asparagine residue (N) of the N‐glycosylation site is underlined. (b) MS2 fragmentation spectrum of the double charged [M + 2H]^2+^ glycopeptide 3 at m/z 1636.27 as illustration. For M3XFGN2 structure, see Figure 4. M, mannose; X, xylose; F, fucose; GN, N‐acetylglucosamine.

The *N*‐glycan profile of rIDUA_RLT has been determined by mass spectrometry analysis of oligosaccharides released from the purified recombinant enzyme (Figure 4). The only ion that can be assigned to glycan residues is the ion at m/z 704.81which corresponds to a *N*‐glycan composed of a core Man_3_GlcNAc_2_ carrying one beta(1,2)‐xylose and one alpha(1,3)‐fucose epitope (M_3_XFGN_2_), a *N*‐glycan widely described in plants (Bardor, 2008; Brooks, 2011; Schoberer and Strasser, 2017). The other ions correspond to non‐glycan contaminants. A unique paucimannosidic *N‐*glycan was thus detected. These results were also confirmed using western blot against beta(1,2)‐xylose and alpha(1,3)‐fucose (data not shown). In order to determine the distribution of this oligosaccharide on each *N*‐glycosylation site of the recombinant rIDUA_RLT secreted by the hairy root clones, a glycoproteomic approach combined to nano LC‐nanoESI‐MS was used as previously described (Vanier *et al*., 2015). This has made it possible to access both to the *N*‐glycan structures and to their site‐occupancy. For that, LC peaks giving MS^2^ spectra exhibiting *N*‐glycan diagnostic fragment ions at *m/z* 204 (*N*‐acetylglucosamine), 163 (mannose) and 366 (Man‐GlcNAc) were assigned to glycopeptides (Figure 5a). Using such assignment and as illustrated in Figure 5a, M_3_XFGN_2_ *N*‐glycan was identified attached to each of the 6 *N*‐glycosylation sites. Sequences of glycopeptides were then confirmed by analysis of the fragmentation patterns obtained by MS^2^ (Figure 5b). The Figure 5 exemplifies the results obtained from the analysis of the rIDUA_RLT produced in one bioreactor batch.

A deglycosylation experiment definitively confirmed that all *N‐*glycosylation sites of the rIDUA_RLT protein are occupied. Indeed, non‐glycosylated peptides containing an Asn residue were not detected in the rIDUA_RLT which indicates that the six sites are fully *N*‐glycosylated. Thus, this result indicates that this hairy root based platform is able to *N*‐glycosylate efficiently the complex recombinant proteins.

### *O*‐glycosylation analysis of rIDUA_RLT produced in the hairy root based expression platform

Like *N*‐linked glycans, *O*‐linked glycans play an important role in protein stability and function (Kim *et al*., 2016; Zhang *et al*., 2013). In order to further characterize the glycosylation profile of the rIDUA_RLT protein produced, an analysis of its *O*‐glycosylation profile was performed.

**Figure 6:**
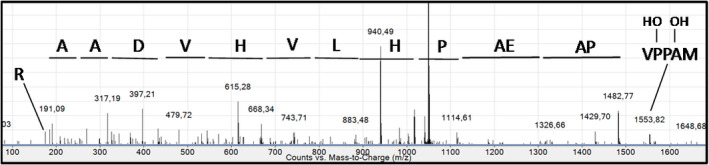
MS2 fragmentation spectrum of the double charged [M + 2H]^2+^ MAPPVAPAEAPHLVHVDAAR N‐terminal peptide (m/z 1049.03) containing two hydroxyproline residues.

As mentioned above, the *N*‐terminal sequencing using Edman degradation displayed a MAXXV sequence at the *N*‐terminal end of the rIDUA_RLT protein. It thus appears that the X residues in the rIDUA_RLT protein may correspond to proline residues that experienced post‐translational modifications. To investigate which modifications may occur on the proline residues at the *N*‐terminal extremity of the rIDUA_RLT protein, a nano LC‐nanoESI‐MS analysis of the recombinant rIDUA_RLT was carried out as described above after tryptic digestion of the recombinant protein. Native *N*‐terminal MAPPVAPAEAPHLVHVDAAR tryptic peptide was not found. However, *N*‐terminal peptides were detected with m/z shifts of 16, 32 and 48 mass units. The sequences of these *N*‐terminal peptides were investigated by MS^2^ sequencing. MS^2^ spectrum of the double charged ion corresponding to the *N*‐terminal peptide exhibiting a m/z shift of 32 mass units is presented in Figure 6. The fragmentation pattern indicated that the m/z shift is carried out by the *N*‐terminal MAPPV amino acid sequence. Considering the absence of detection of proline residues in the *N*‐terminal Edman degradation, we thus conclude that these two proline residues are hydroxylylated in the rIDUA_RLT produced in *B. rapa* hairy roots. As well, peptides with m/z shifts of 16 and 48 mass units were assigned to *N*‐terminal sequences containing one and three hydroxyproline residues, respectively. Based on the ion intensity, the relative percentage of the *N*‐terminal peptide bearing one hydroxyproline is about 40%, the one of the peptide bearing two hydroxyproline residues represent almost 60% and *N*‐terminal peptide containing 3 hydroxyproline residues represent about 1%.

In addition to m/z shifts resulting from the hydroxylation of proline residues, *N*‐terminal MAPPVAPAEAPHLVHVDAAR tryptic peptides with m/z shifts of 132 or 264 and representing about 1% of the overall peptide population were also detected. These peptides may result from the transfer of pentose residues onto hydroxyproline residues as observed in plants (Schoberer and Strasser, 2017). To determine which pentose may be linked to hydroxyproline residues, a monosaccharide composition of rIDUA_RLT was determined by gas chromatography analysis (Table 1). Xylose, fucose, GlcNAc and mannose were detected which is consistent with the *N*‐glycosylation profile of this protein as depicted in Figures 4 and 5. In addition, arabinose residues, and no other pentose, were also detected suggesting that the pentose *O*‐linked to hydroxyproline is indeed arabinose (Table 1). Glucose and galactose are contaminant residues.

**Table 1:** Monosaccharide composition of alpha‐L‐iduronidase produced in *Brassica rapa* hairy roots

Monosaccharide	Relative molar composition %
Arabinose (Ara)	7.2
Fucose (Fuc)	5.3
Xylose (Xyl)	7.6
Mannose (Man)	39.3
Galactose (Gal)	18.7
Glucose (Glc)	2.1
N‐acetylGlucosamine (GlcNAc)	19.8

A second peptide containing a PPXP sequence (peptide STGFCPPLPHSQADQYVLSWDQQLNLAYVGAVPHR) was identified as being hydroxylated. One to three hydroxylations were detected on this peptide by LC‐ESI‐MS. In addition to mono‐, di‐ and trihydroxylated forms of the peptide, a tetrahydroxylated variant was also detected by nanoLC‐nanoESI‐MS.

### *N/O*‐glycosylation homogeneity and reproducibility of rIDUA_RLT

Using the same analytical strategy, searches for other posttranslational modifications, such as phosphorylation of hydroxy aminoacids, were unsuccessful. In addition, similar results were obtained on the analysis of the *N*‐ and *O‐*glycosylation of the rIDUA_RLT protein produced at 6‐month intervals in two independent bioreactor batches (data not shown). On each of the 6 *N*‐glycosylation sites of both rIDUA_RLT proteins produced using the two independent bioreactor batches, a single *N*‐glycan composed of a core Man_3_GlcNAc_2_ carrying one beta (1,2)‐xylose and one alpha(1,3)‐fucose epitope (M3XFGN2) was identified. Moreover, the same hydroxylation and arabinosylation modifications were identified on both rIDUA_RLT during *O‐*glycosylation analysis. These results indicate that expression of rIDUA_RLT in hairy roots is robust and reproducible not only in term of protein productivity (Figure 1c) but also in term of *N*‐ and *O*‐glycosylation profiles.

## Discussion

In the present work, the ability of the *B. rapa* hairy root based expression platform to produce and secrete a complex recombinant glycoprotein in its active form was demonstrated taking as an example the rIDUA_RLT protein. This highlights the relevance of the platform as an expression system. It was shown that the hairy‐root based rIDUA_RLT protein shows enzymatic characteristics similar to the ones of the same recombinant protein produced in CHO (Aldurazyme) despite the differences that exist between both expression systems in terms of post‐translational modifications.

The *N*‐glycosylation analysis of rIDUA_RLT showed that the recombinant protein displays a single paucimannose profile on all *N*‐glycosylation sites. Such a homogeneity is remarkable because in most expression systems commonly used for the production of biopharmaceuticals, *N*‐glycosylation of recombinant proteins carry multiple *N*‐glycan structures resulting from the variability of *N*‐glycan maturation occurring in the Golgi apparatus. This results in recombinant therapeutic proteins exhibiting high heterogeneity which may affect the batch‐to‐batch reproducibility (Hossler *et al*., 2009; Lingg *et al*., 2012). As an example, Aldurazyme, the rIDUA protein produced in CHO cells, is characterized by a high intra‐site heterogeneity of the *N*‐linked glycans and this was observed in each of the six *N*‐glycosylation sites (Zhao *et al*., 1997). Indeed, the site specific glycosylation pattern that characterize Aldurazyme is: Asn‐110, complex type glycans; Asn‐190, complex type glycans; Asn‐336, bisphosphorylated oligomannosidic glycan (P2Man7GlcNAc2); Asn‐372, high mannose type glycans (mainly Man9GlcNAc2, some of which are monoglucosylated); Asn‐415, mixed oligomannosidic and complex type glycans; Asn‐451, bisphosphorylated oligomannosidic glycan (P2Man7GlcNAc2; Zhao *et al*., 1997); which contrasts with the single paucimmanose profile found in all N‐glycosylation sites of rIDUA_RLT. Similarly, the recombinant IDUA proteins produced in other plant‐based expression systems than the hairy roots are characterized by a heterogeneity of the N‐glycosylation profiles with nevertheless a predominance of high mannose profiles (He *et al*., 2012, 2013). The absence of such a large heterogeneity when analysing the *N‐*glycosylation profiles of the rIDUA_RLT produced using the *B. rapa* hairy root system is of particular interest at a regulatory level to increase the reproducibility of the batches that may be used in clinical trials.

Such observation was also made when analysing total endogenous proteins of *B. rapa* hairy root clones developed using the hairy root platform. As an example, total endogenous proteins from isolated young or old roots collected at different time‐points of the culture of hairy root clones expressing the glucocerebrosidase (GCD) recombinant protein still essentially display profiles of paucimannosidic type (see Data S1 and Figure S1) when analysed by mass spectrometry, reinforcing our observation. High‐mannose *N*‐glycans were not detected in the oligosaccharide profile. We postulate that *B. rapa* Golgi α‐mannosidases are likely highly efficient in the processing of high‐mannose *N*‐glycans arising from Endoplasmic Reticulum biosynthesis steps into mature glycans. These additional observations reinforce the demonstration of the ability of the *B. rapa* hairy root platform to produce recombinant proteins with a remarkable homogeneous glycosylation profile, never observed in the recombinant proteins produced in CHO (Tekoah *et al*., 2013; Zhao *et al*., 1997).

As in mammals, plants can also produce proteins displaying O‐glycosylation profiles (Tekoah, 2006). However, the O‐glycosylation profiles of the recombinant proteins produced from plant‐based expression systems were only poorly described in the literature (Karnoup *et al*., 2005; Kim *et al*., 2016; Schoberer and Strasser, 2017). *O*‐linked glycosylation can be found in amino acids that contain a hydroxyl group (i.e. serine, threonine, hydroxyproline and hydroxylysine). Hydroxylation of the proline amino acids takes place in the Endoplasmic Reticulum, then O‐glycosylation occurs in the Golgi apparatus (Schoberer and Strasser, 2017). In our study, hydroxylation and *O*‐glycosylation of proline residues have been highlighted on rIDUA_RLT. Such hydroxylation was already observed in the recombinant IDUA protein produced in CHO (Jung *et al*., 2013). However, to the best of our knowledge, no analysis of the *O*‐glycan profiles associated with this protein was further investigated although this mechanism is indeed present in mammals. *O*‐glycosylation has also been described in plants, where it converts the proline residues to hydroxyproline and attaches arabinose residues to recombinant proteins (Karnoup *et al*., 2005; Pinkhasov *et al*., 2011). In the monosaccharide composition of rIDUA_RLT, in addition to xylose of *N*‐glycans, only one pentose, arabinose, was observed suggesting that the m/z shifts of 132 of hydroxyproline‐containing peptides are indeed due to arabinose attachment.

Finally, the rIDUA_RLT presents a similar glycan profile as the one classically observed in proteins produced by other plant‐based expression systems, in particular the well‐characterized beta(1,2)‐xylose and one alpha(1,3)‐fucose epitopes. These residues have been criticized likely as being immunogenic (Bardor *et al*., 2003). However, this observation is still controversial and depends on the studied model. Elelyso, a recombinant GCD produced using carrot cells by Protalix (Carmiel, Israel) and used in clinic since 2012 is a good example of the non‐toxicity of such glycoepitopes. Indeed, Elelyso is characterized by the presence of the plant‐specific residues, i.e. beta(1,2)‐xylose and alpha(1,3)‐fucose. In addition, signals consistent with the presence of arabinose were detected in the monosaccharide composition of the carrot cells‐produced GCD (Shaaltiel *et al*., 2015). Nevertheless, no adverse effect was observed that could be linked to such particular residues in all patients treated so far using this therapeutic product, neither during the clinical trials, nor since its approval by the regulatory authorities. This plant‐based protein appeared as safe as the treatment of the patients using the counterpart protein produced in CHO (Cerezyme^®^/Sanofi) or human fibroblast cells (VPRIV^®^/Shire Dublin, Ireland; Shaaltiel and Tekoah, 2016). The glycosylation profile of the recombinant proteins produced using the *B. rapa* hairy root platform could be thus compatible with a therapeutic use of such proteins.

Finally, thanks to the highly homogeneous paucimannosidic profile of its recombinant proteins, the *B. rapa* hairy root based expression platform is of particular relevance for the production of proteins of therapeutic interest such as the GCD for the treatment of Gaucher disease or the alpha galactosidase for the treatment of the patients with Fabry disease. Regarding the treatment of other lysosomal disorders, the addition of mannose‐6‐phosphate (M6P) residues would be ideally required *in vivo* as the plants are not naturally able to phosphorylate the mannose residue*s*. Several strategies are described in the literature allowing the addition of such residues on plant‐based recombinant proteins (He *et al*., 2012, 2013). The existence of alternate M6P‐independent pathways for lysosomal enzyme sorting has also been largely described (Markmann *et al*., 2015). As an example, based on Kakkis results (Kakkis *et al*., 1994), 20% of the IDUA protein is able to penetrate the targeted cell through an alternative pathway to the well‐characterized M6P recognition system. This independent glycosylation pathway can be related to the usage of alternatives routes enabling recombinant proteins to penetrate the targeted cells such as the use of HIV Tat peptides (Xia *et al*., 2001; Zhang *et al*., 2008), insulin growth factor II (LeBowitz *et al*., 2004), receptor associated protein RAP (Prince *et al*., 2004), insulin receptor (Boado *et al*., 2008, Lu *et al*., 2011), intercellular adhesion molecule 1 (ICAM‐1; Hsu *et al*., 2011, Hsu *et al*., 2012, Muro *et al*., 2006), transferrin receptor (Tfr; Chen *et al*., 2008, Osborn *et al*., 2008), RTB lectin (Acosta *et al*., 2015).

As an example, an α‐glucosidase (GAA) recombinant protein, enzyme involved in Pompe disease, coupled to polymer nanocarriers coated with an antibody specific to ICAM‐1, allowed the efficient internalization and lysosomal transport of GAA into target cells enhancing substrate degradation (Hsu *et al*., 2012). In the same way, a galactocerebrosidase tagged with HIV Tat protein facilitated its uptake into neurons using M6P independent pathway (Zhang *et al*., 2008). More recently, Sonoda *et al*. (2018) showed that the recombinant iduronate‐2‐sulphatase fused with an anti‐TfR antibody was able to be uptaken by fibroblastes through both the TfR and the M6P pathways. All these examples show that several alternative strategies can be used to enhance the uptake of recombinant lysosomal enzymes, including alpha‐L‐Iduronidase, even if the N‐glycan phosphorylation is lacking in these recombinant proteins.

## Material and methods

### Materials

*Escherichia coli* strain JM101 and *R. rhizogenes* strain ICPB TR7 were used for cloning and plant transformation, respectively, and *B. rapa rapa* cv ‘Navet des vertus marteau’ for hairy root production. Plant tissue culture media, vitamins and sucrose came from Duchefa Biochemie. 4‐methylumbelliferyl‐α‐L‐Iduronide (4MU‐I) came from Santa Cruz Biotechnology (Dallas, TX). The commercial recombinant IDUA protein used as positive control came from Antibodies‐online. The anti‐IDUA antibody used in the Western‐blot analyses came from Antibodies‐online. All reagents used to study the post‐translational modifications of the IDUA protein were of HPLC grade. Peptide *N*‐Glycosidase A was purchased from Roche Mannheim, Germany. All reagents used for SDS‐PAGE silver staining, ‐Methylumbelliferone (4MU) and Concanavalin A were purchased from Sigma (Saint‐Louis, MO). Antibodies directed against the anti‐xylose and anti‐fucose epitopes were from Agrisera Vännäs, Sweden.

### Molecular cloning

Gene synthesis of the human Iduronidase A (IDUA) (NCBI NP_000194.2) coding sequence was performed by GeneART (ThermoFisher Scientific, Regensburg, Germany) including the Tobacco Mosaic Virus (TMV) omega translational enhancer, the signal peptide (SP) coding sequence from the *Arabidopsis* At1g69940*pme* gene and the *HindIII* and *EcoRI* restriction sites for easy subcloning into the previously described pJIT163 plasmid (Guerineau, 1995). The expression cassette containing the omega translational enhancer, the SP and the IDUA sequence was cloned into *HindIII*/*EcoRI* restriction sites of the binary plant expression vector pRD400 (Datia *et al*., 1992) containing an upstream 35S Cauliflower Mosaic Virus (CaMV) promoter and a downstream CaMV 35S terminator. The pRD400 vector was then inserted in *R. rhizogenes* bacteria.

### Transgene expression detection

Total RNA extracts from 0.1 g transformed fresh roots were prepared with the kit Total RNA and Protein Isolation (Macherey‐Nagel, Düren, Germany). 0.1 μg/μL of total RNA extracts were used to generate the cDNA by using the M‐MuLV Reverse Transcriptase (New England Biolabs, Ipswich, MA). The cDNA was then amplified using the specific primers: 5′‐TTCTGTCCTCCTCTCCCTCA‐3′, 5′‐AGGGACCTCTAAGTACGGCA‐3′ for hIDUA and 5′‐ATTCCGTCGTCGATCCTCT‐3′, 5′‐ACCGACGATGATGTTGTTGA 3′ for SEC61.

### Plant transformation and hairy root culture

Turnip plants were transformed as described in (Huet *et al*., 2014). Briefly, after 10 days growth, the elongated stems originating from the culture of Turnip (*B. rapa L. var. rapa cv. des vertus marteau*) seeds were infected using the *R. rhizogenes* prepared as described above. The roots emerging from the infection sites were individualized and placed on medium B5 Gamborg (Gamborg *et al*., 1968). Isolated root lines were screened according to their growth capacity and their ability to produce the rIDUA_RLT protein by Western‐blot analysis. The culture was carried out as described previously (Ele Ekouna *et al*., 2017). For pilot scale production, root clones were cultured in 25 L airlift bioreactors.

### Protein analysis

#### Western blot

Samples (crude culture media) were resolved in AnykD mini protean TGX polyacrylamide gels (Bio‐Rad). For Western blot analysis, proteins were transferred to nitrocellulose membranes (Bio‐Rad, Hercules, California) using the Bio‐Rad Turbo Trans‐Blot system. The membranes were blocked in 5% fat‐free milk (Blotting grade blocker, Bio‐Rad) in TBS buffer, incubated with a 1:1000 dilution of the mouse anti‐α‐L‐Iduronidase (ABIN603316 from Antibodies‐online) followed by a 1:5000 dilution of a goat anti‐mouse IgG‐HRP antibody (sc‐2005 from Santa Cruz Biotechnology). Staining was developed using Western Clarity ECL revelation kit (170‐5060, Bio‐Rad).

#### Silver staining of proteins in polyacrylamide gels

Samples (crude culture media) or purified protein were resolved in AnykD mini protean TGX polyacrylamide gels (BioRad). For silver stain, gels were incubated in 50% ethanol/10% acetic acid during 30 min, then in 30% ethanol/1% acetic acid for 15 min. Gels were washed in ultrapure H_2_O with shaking, three times for 10 min. Gels were sensitized during 90 s in 0.02% sodium thiosulphate, 6.8% sodium acetate then rinsed in H_2_O. Gels were incubated in 0.1% silver nitrate for 10 min. Gels were incubated in 3% sodium carbonate, 0.0002% sodium thiosulphate and 0.025% formaldehyde with shaking during 1 min. Revelation were stopped with a 40 mm EDTA‐Na_2_ solution for 5 min.

#### MS analysis

For nanoLC/MS/MS analysis, samples were prepared as described in (Allmann *et al*., 2014). The peptides were analysed on an Ultimate 3000 RSLC Nano‐UPHLC system (Thermo Scientific, Waltham, Massachusetts) coupled to a nanospray Q‐Exactive hybrid quadrupole‐Orbitrap mass spectrometer (Thermo Scientific). Ten microliters of each peptide extract were loaded onto a 300 μm ID × 5 mm PepMap^®^ C18 pre‐column (Thermo Scientific) at a flow rate of 20 μL/min. After 5 min desalting, peptides were separated on a 75 μm ID × 25 cm C18 Acclaim PepMap^®^ RSLC column (Thermo Scientific) with a 4%–40% linear gradient of solvent B (0.1% formic acid in 80% CH_3_CN) in 108 min. The separation flow rate was set at 300 nL/min. The mass spectrometer was operated in positive ion mode at a needle voltage of 1.8 kV. Data were acquired using Xcalibur 3.1 software in a data‐dependent mode. MS scans (m/z 350–1600) were recorded at a resolution of *R* = 70 000 (@ m/z 200) and an AGC target of 3 × 10^6^ ions was collected within 100 ms. Dynamic exclusion was set at 30 s and the top 12 ions were selected from fragmentation in HCD mode. MS/MS scans with a target value of 1 × 10^5^ ions were collected with a maximum fill time of 100 ms and a resolution of *R* = 17 500. Additionally, only +2 and +3 charged ions were selected for fragmentation. Other settings were as follows: no sheath and no auxiliary gas flow, capillary heated at 200 °C, normalized HCD collision energy of 27% and isolation width of 2 m/z.

Mascot, Sequest and Amanda algorithms through Proteome Discoverer 1.4 Software (Thermo Fisher Scientific Inc.) were used for protein identification in batch mode by searching against a merge of protein databases: UniProt *Arabidopsis thaliana* database (31 587 entries, Reference Proteome Set, release 2015_01) and UniProt *Brassica Rapa subsp*. pekinensis database (40 809 entries, release 2015_01) from http://www.uniprot.org/ website and the sequence of the recombinant protein. Two missed enzyme cleavages were allowed. Mass tolerances in MS and MS/MS were set at 10 ppm and 0.02 Da. Peptide validation was performed using the Percolator algorithm (Kall *et al*., 2007) and only high confidence peptides were retained corresponding to a 1% False Positive Rate at peptide level.

### rIDUA_RLT purification

Culture medium was collected, clarified by centrifugation at 9000 *g* for 20 min at 4 °C and filtered through two successive steps using a 0.8–0.45 μm and a 0.45–0.2 μm filters (Sartopore 2 Midicap). The fraction was applied on the strong cation exchanger chromatography Eshmuno S from Millipore equilibrated with sodium acetate 100 mm, urea 1.5 m, pH 5.0 followed by the same buffer without urea 1.5 m. The elution step was performed at 25 mS/cm with 20% (v/v) sodium acetate 100 mm, NaCl 1 m pH 5.0 followed by a step at 34 mS/cm with 30% (v/v) of the same buffer. The fractions containing the rIDUA_RLT protein were collected and applied on a hydrophobic interaction chromatography (HIC) resin Toyopearl Phenyl 650 m from Tosoh equilibrated with 20 mm sodium phosphate, pH 7.0 with and without 3 m NaCl. The first step of elution was performed with 30% (v/v) of 20 mm sodium phosphate containing 3 m NaCl pH 7.0. A gradient from 35% to 100% of sodium phosphate 20 mm, NaCl 3 m pH7.0 was then applied in order to recover the rIDUA_RLT protein. The pooled fractions containing rIDUA_RLT were concentrated using a membrane cut‐off of 30 kDa (Sartocon cassette PES – Sartorius, Göttingen, Germany) and submitted to a diafiltration using 10 volumes of buffer NaH_2_PO_4_.2H_2_O 92 mm, Na_2_HPO_4_.12H_2_O 8 mm, NaCl 150 mm, pH 6.0 to achieve a protein concentration of 0.58 mg/L. Tween 80 was finally added to achieve a final concentration of 10 mg/L. The material collected from the formulation step was submitted to a 0.2 μm filtration using a Minisart PES filter, aliquoted and stored at −20 °C. Quality of the purified material and concentration of the pure enzyme was determined by SDS‐PAGE, western‐blot, SE‐HPLC and RP‐HPLC.

#### SE‐HPLC analysis

The purity level of IDUA protein as well as the presence of soluble aggregates were analysed using a SE‐HPLC method performed on a HPLC Agilent system (HPLC Agilent Series 1200 Infinity) equipped with a refrigerated autosampler, a quaternary pump, a column heater and a DAD detector. A volume of 100 μL of sample was injected onto a Superdex 200 Increase 10/300 column (GE). The mobile phase was PBS, pH 7.2 buffer (NaCl, 8 g/L, KCl 0.2 g/L, Na_2_HPO_4_.12 H_2_O 3.58 g/L, KH_2_P0_4_ 0.24 g/L). The flow rate was 0.5 mL/min. The SE‐HPLC profile was recorded at 280 nm.

### Determination of the human α‐L‐Iduronidase activity

Turnip hairy root culture media coming from transformed hairy root cultures as well as the purified rIDUA_RLT protein were used to determine the activity of the recombinant protein of interest by using the fluorogenic substrate sodium 4‐methylumbelliferyl‐α‐L‐Iduronide (4MU‐I; Santa Cruz Biotechnology) as described in (Ou *et al*., 2014). The 4MUI substrate was diluted to a working solution of 400 μm 4MU‐I with the reaction buffer 0.4 m sodium formate, pH 3.5. Twenty‐five μL of sample were added to 25 μL of 400 μm 4MU‐I substrate. The mixture was incubated at 37 °C for 30 min and 200 μL glycine carbonate buffer (pH 9.8) was added to quench the reaction. 4‐Methylumbelliferone (4MU) (Sigma) was used to prepare the standard calibration curve. Fluorescence was measured using a plate reader (TECAN Infinite M1000, Männedorf, Switzerland) with excitation at 355 nm and emission at 460 nm. IDUA enzyme activity was expressed in units (μmol converted to product per minute) per sample volume (millilitres). The parameters *K*_M_, *k*_cat_ and *V*_max_ were calculated by linear fit on a Lineweaver‐Burk plot (Ou *et al*., 2014).

### Determination of the *N*‐Terminal sequence

The rIDUA_RLT produced and purified from hairy roots culture medium was separated by an 8% SDS‐PAGE, and then transferred to a PVDF membrane using the ProSorb system from Applied Biosystems. *N*‐terminal sequence was then determined automatically by Edman degradation with the Procise P494 (Applied Biosystem, Foster City, California).

### Analysis of the glycosylation of the purified rIDUA_RLT

#### Identification of the total *N*‐glycan profile of rIDUA_RLT

rIDUA_RLT was digested with proteases prior to a deglycosylation using the PNGase A as previously described (Baïet *et al*., 2011, Mathieu‐Rivet *et al*., 2013). Released *N*‐glycans were then purified over C18 and PGC pre‐packed columns (Bakker *et al*., 2001, Ho *et al*., 2012). Finally, labelling of the purified *N*‐glycans to procainamide (proc) was carried out by reducing amination according to the manufacturer instructions (Ludger LTD, Culham, Oxfordshire, UK LudgerTag Procainamide Glycan Labeling Kit). Procainamide derivatized *N*‐glycans were then analysed by nanoLC coupled to nanoESI‐MS using the nano‐LC1200 system coupled to a QTOF 6520 mass spectrometer equipped with a nanospray source and a LC‐Chip Cube interface (Agilent Technologies, Santa Clara, California).

#### Sample preparation

Purified rIDUA_RLT was separated on a NuPAGE Bis‐Tris gel electrophoresis. Band corresponding to rIDUA_RLT was excised from the gel and cut into pieces. Gel pieces were washed several times with a solution mixture composed of 0.1 m NH_4_HCO_3_ pH 8 and 100% CH_3_CN (v: v). Samples were dried down in a SpeedVac centrifuge (Thermo Fisher). After a reduction step with 0.1 m dithiothreitol (DTT) for 45 min at 56 °C and alkylation with 55 mm iodoacetamide (IAA) for 30 min at room temperature in the dark, proteomic‐grade trypsin was added (1 μg per protein band; Promega) and placed at 4 °C during 45 min prior to an overnight incubation at 37 °C. Then, rIDUA_RLT was eventually submitted to an additional chymotrypsin digestion. After protease digestion, gel pieces were incubated subsequently in a 50% CH_3_CN solution, 5% formic acid solution, 0.1 m NH_4_HCO_3_, 100% CH_3_CN and finally in 5% formic acid to extract the resulting peptide and glycopeptide mixture.

#### Protein identification and site‐specific distribution of *N‐* and *O*‐glycans

MS analyses were performed using the nano‐LC1200 system coupled to a QTOF 6520 mass spectrometer equipped with a nanospray source and a LC‐Chip Cube interface (Agilent Technologies). Briefly, peptide and glycopeptide mixture was enriched and desalted on a 360 nL RP‐C18 trap column and separated on a Polaris (3‐μm particle size) C18 column (150 mm long × 75 μm inner diameter; Agilent Technologies). A 33‐min linear gradient (3%–75% acetonitrile in 0.1% formic acid) at a flow rate of 320 nL/min was used, and separated peptides were analysed with the QTOF mass spectrometer. Full auto MS scans from 290 to 1700 *m/z* and auto MS^2^ from 59 to 1700 *m/z* were recorded. In every cycle, a maximum of 5 precursors sorted by charge state (2^+^ preferred and single‐charged ions excluded) were isolated and fragmented in the collision cell. Collision cell energy was automatically adjusted depending on the *m/z*. Scan speed raise based on precursor abundance (target 25 000 counts/spectrum) and precursors sorted only by abundance. Active exclusion of these precursors was enabled after three spectra within 1.5 min, and the threshold for precursor selection was set to 1000 counts. Glycopeptides were selected in the LC profile by selecting MS^2^ spectra exhibiting *N*‐glycan diagnostic fragment ions at *m/z* 204 and 163. Mass hunter qualitative analysis version B.07 (Agilent Technologies) was used to analyse the spectra.

In order to evaluate the site occupancy, IDUA peptides and glycopeptides mixture was deglycosylated by using the peptide N‐glycosidase A (PNGase A; Roche diagnostic) according to (Vanier *et al*., 2015).

#### Monosaccharide composition using gas chromatography

The monosaccharide composition was determined by gas chromatography using 100 nmol of inositol as internal standard. rIDUA_RLT was hydrolysed during 2 h in 2 m TFA (trifluoroacetic acid) at 110 °C. Freeze‐dried samples were methanolysed with dry 1 m methanolic‐HCl (Supelco) for 16 h at 80 °C and then were dried under a stream of nitrogen and washed twice with methanol. Re‐*N*‐acetylation step was performed in methanol/pyridine/acetic anhydride 4:1:1 (Sigma) for 1 h at 110 °C. The resulting methyl sugars were treated with HDMS (hexamethyldisilazane)/TMCS (trimethylchlorosilane)/pyridine solution (3:1:9, Supelco), for 20 min at 110 °C. Trimethylsilylated monosaccharides were dried and dissolved in 1 mL of cyclohexane. One μl of sample was separated by gas chromatography on a 0.25 mm × 25 m silica capillary column of CP‐Sil 5 CB with helium as a carrier gas and detected with a flame ionization detector. Neutral sugar standards (L‐arabinose, L‐fucose, D‐galactose, D‐mannose, D‐galacturonic acid, L‐rhamnose, D‐glucose, D‐GlcNAc, and D‐xylose) were processed in parallel to the samples. Assignment of sample peaks was carried out by comparison of their retention times with those of standards treated and analysed in parallel.

## Conflict of interest

Florian Cardon, Roser Pallisse, Aurore Caron and Marina Guillet are employees of Root Lines Technology SA, the sponsor of this study, and as such have vested commercial interests.

## Supporting information


**Figure S1** ESI mass spectrum of N‐glycans isolated from endogenous proteins of old roots collected at day 24.

**Data S1** Methodological details for the analysis of the glycosylation of endogenous protein as well as the analysis of the purity of the protein of interest.

## References

[pbi12994-bib-0001] Acosta, W. , Ayala, J. , Dolan, M.C. and Cramer, C.L. (2015) RTB Lectin: a novel receptor‐independent delivery system for lysosomal enzyme replacement therapies. Sci. Rep. 5, 14144. 26382970 10.1038/srep14144 PMC4585660

[pbi12994-bib-0002] Allmann, S. , Mazet, M. , Ziebart, N. , Bouyssou, G. , Fouillen, L. , Dupuy, J.‐W. , Bonneu, M. *et al*. (2014) Triacylglycerol storage in lipid droplets in Procyclic *Trypanosoma brucei* . PLoS ONE, 9, e114628. 25493940 10.1371/journal.pone.0114628 PMC4262433

[pbi12994-bib-0003] Baïet, B. , Burel, C. , Saint‐Jean, B. , Louvet, R. , Menu‐Bouaouiche, L. , Kiefer‐Meyer, M.‐C. , Mathieu‐Rivet, E. *et al*. (2011) N‐glycans of phaeodactylum tricornutum diatom and functional characterization of its N‐acetylglucosaminyltransferase I enzyme. J. Biol. Chem. 286, 6152–6164. 21169367 10.1074/jbc.M110.175711 PMC3057864

[pbi12994-bib-0004] Bakker, H. , Bardor, M. , Molthoff, J.W. , Gomord, V. , Elbers, I. , Stevens, L.H. , Jordi, W. *et al*. (2001) Galactose‐extended glycans of antibodies produced by transgenic plants. Proc. Natl. Acad. Sci. USA, 98, 2899–2904. 11226338 10.1073/pnas.031419998 PMC30237

[pbi12994-bib-0005] Bardor, M. (2008) Plant N‐glycosylation: an engineered pathway for the production of therapeutical plant‐derived glycoproteins. Comp. Biochem. Physiol. A: Mol. Integr. Physiol. 150, S164.

[pbi12994-bib-0006] Bardor, M. , Faveeuw, C. , Fitchette, A.C. , Gilbert, D. , Galas, L. , Trottein, F. , Faye, L. *et al*. (2003) Immunoreactivity in mammals of two typical plant glyco‐epitopes, core alpha(1,3)‐fucose and core xylose. Glycobiology, 13, 427–434. 12626420 10.1093/glycob/cwg024

[pbi12994-bib-0008] van Beers, M.M.C. and Bardor, M. (2012) Minimizing immunogenicity of biopharmaceuticals by controlling critical quality attributes of proteins. Biotechnol. J. 7, 1473–1484. 23027660 10.1002/biot.201200065

[pbi12994-bib-0009] Boado, R.J. , Zhang, Y. , Xia, C.F. , Wang, Y. and Pardridge, W.M. (2008) Genetic engineering of a lysosomal enzyme fusion protein for targeted delivery across the human blood‐brain barrier. Biotechnol. Bioeng. 99, 475–484. 17680664 10.1002/bit.21602

[pbi12994-bib-0010] Brooks, S.A. and Society and Biology, F. E. (2011) Glycosylation in diverse cell systems: challenges and new frontiers in experimental biology. Essent. Rev. Exp. Biol. 4, 93–118.

[pbi12994-bib-0011] Chen, C.H. , Dellamaggiore, K.R. , Ouellette, C.P. , Sedano, C.D. , Lizadjohry, M. , Chernis, G.A. , Gonzales, M. *et al*. (2008) Aptamer‐based endocytosis of a lysosomal enzyme. Proc. Natl. Acad. Sci. USA, 105, 15908–15913. 18838694 10.1073/pnas.0808360105 PMC2572987

[pbi12994-bib-0012] Datia, R.S.S. , Hammerlindl, J.K. , Panchuk, B. , Pelcher, L.E. and Keller, W. (1992) Modified binary plant transformation vectors with the wild‐type gene encoding NPTII. Gene, 122, 383–384. 1336757 10.1016/0378-1119(92)90232-e

[pbi12994-bib-0013] Dudek, J. , Pfeffer, S. , Lee, P.H. , Jung, M. , Cavalie, A. , Helms, V. , Forster, F. *et al*. (2015) Protein transport into the human endoplasmic reticulum. J. Mol. Biol. 427(6 Pt A), 1159–1175. 24968227 10.1016/j.jmb.2014.06.011

[pbi12994-bib-0014] Ele Ekouna, J.‐P. , Boitel‐Conti, M. , Lerouge, P. , Bardor, M. and Guerineau, F. (2017) Enhanced production of recombinant human gastric lipase in turnip hairy roots. Plant Cell Tissue Organ Cult. 131, 601–610.

[pbi12994-bib-0017] Gamborg, O.L. , Miller, R.A. and Ojima, K. (1968) Nutrient requirements of suspension cultures of soybean root cells. Exp. Cell Res. 50, 151–158. 5650857 10.1016/0014-4827(68)90403-5

[pbi12994-bib-0018] Georgiev, M.I. , Pavlov, A.I. and Bley, T. (2007) Hairy root type plant *in vitro* systems as sources of bioactive substances. Appl. Microbiol. Biotechnol. 74, 1175. 17294182 10.1007/s00253-007-0856-5

[pbi12994-bib-0019] Giri, A. and Narasu, M.L. (2000) Transgenic hairy roots. Biotechnol. Adv. 18, 1–22. 14538116 10.1016/s0734-9750(99)00016-6

[pbi12994-bib-0020] Guerineau, F. (1995) Tools for expressing foreign genes in plants. In Plant Gene Transfer and Expression Protocols ( Jones, H. , ed), pp. 1–32. Totowa, NJ: Springer New York. 10.1385/0-89603-321-X:1 8563796

[pbi12994-bib-0021] Guillon, S. , Tremouillaux‐Guiller, J. , Pati, P.K. , Rideau, M. and Gantet, P. (2006a) Hairy root research: recent scenario and exciting prospects. Curr. Opin. Plant Biol. 3, 341–346. 10.1016/j.pbi.2006.03.008 16616871

[pbi12994-bib-0022] Guillon, S. , Trémouillaux‐Guiller, J. , Pati, P.K. , Rideau, M. and Gantet, P. (2006b) Harnessing the potential of hairy roots: dawn of a new era. Trends Biotechnol. 24, 403–409. 16870285 10.1016/j.tibtech.2006.07.002

[pbi12994-bib-0023] He, X. , Haselhorst, T. , von Itzstein, M. , Kolarich, D. , Packer, N.H. , Gloster, T.M. , Vocadlo, D.J. *et al*. (2012) Production of α‐L‐iduronidase in maize for the potential treatment of a human lysosomal storage disease. Nat. Commun. 3, 1062. 22990858 10.1038/ncomms2070

[pbi12994-bib-0024] He, X. , Pierce, O. , Haselhorst, T. , von Itzstein, M. , Kolarich, D. , Packer, N.H. , Gloster, T.M. *et al*. (2013) Characterization and downstream mannose phosphorylation of human recombinant α‐L‐iduronidase produced in Arabidopsis complex glycan‐deficient (cgl) seeds. Plant Biotechnol. J. 11, 1034–1043. 23898885 10.1111/pbi.12096 PMC4030584

[pbi12994-bib-0025] Ho, S.C. , Bardor, M. , Feng, H. , Mariati , Tong, Y.W. , Song, Z. , Yap, M.G.S. *et al*. (2012) IRES‐mediated Tricistronic vectors for enhancing generation of high monoclonal antibody expressing CHO cell lines. J. Biotechnol. 157, 130–139. 22024589 10.1016/j.jbiotec.2011.09.023

[pbi12994-bib-0026] Hossler, P. , Khattak, S.F. and Li, Z.J. (2009) Optimal and consistent protein glycosylation in mammalian cell culture. Glycobiology, 19, 936–949. 19494347 10.1093/glycob/cwp079

[pbi12994-bib-0027] Hsu, J. , Serrano, D. , Bhowmick, T. , Kumar, K. , Shen, Y. , Kuo, Y.C. , Garnacho, C. *et al*. (2011) Enhanced endothelial delivery and biochemical effects of alpha‐galactosidase by ICAM‐1‐targeted nanocarriers for Fabry disease. J. Control. Release, 149, 323–331. 21047542 10.1016/j.jconrel.2010.10.031 PMC3073729

[pbi12994-bib-0028] Hsu, J. , Northrup, L. , Bhowmick, T. and Muro, S. (2012) Enhanced delivery of alpha‐glucosidase for Pompe disease by ICAM‐1‐targeted nanocarriers: comparative performance of a strategy for three distinct lysosomal storage disorders. Nanomedicine, 8, 731–739. 21906578 10.1016/j.nano.2011.08.014 PMC3279604

[pbi12994-bib-0029] Huet, Y. , Ekouna, J.P. , Caron, A. , Mezreb, K. , Boitel‐Conti, M. and Guerineau, F. (2014) Production and secretion of a heterologous protein by turnip hairy roots with superiority over tobacco hairy roots. Biotechnol. Lett. 36, 181–190. 24078130 10.1007/s10529-013-1335-y

[pbi12994-bib-0030] Jung, G. , Pabst, M. , Neumann, L. , Berger, A. and Lubec, G. (2013) Characterization of α‐l‐Iduronidase (Aldurazyme^®^) and its complexes. J. Proteomics. 80, 26–33. 23026551 10.1016/j.jprot.2012.09.022

[pbi12994-bib-0031] Kakkis, E.D. , Matynia, A. , Jonas, A.J. and Neufeld, E.F. (1994) Overexpression of the human lysosomal enzyme α‐L‐iduronidase in Chinese hamster ovary cells. Protein Expr. Purif. 5, 225–232. 7950365 10.1006/prep.1994.1035

[pbi12994-bib-0032] Kall, L. , Canterbury, J.D. , Weston, J. , Noble, W.S. and MacCoss, M.J. (2007) Semi‐supervised learning for peptide identification from shotgun proteomics datasets. Nat. Methods, 4, 923–925. 17952086 10.1038/nmeth1113

[pbi12994-bib-0033] Karnoup, A.S. , Turkelson, V. and Anderson, W.H. (2005) O‐linked glycosylation in maize‐expressed human IgA1. Glycobiology, 15, 965–981. 15901675 10.1093/glycob/cwi077

[pbi12994-bib-0034] Kim, J. , Park, H. , Park, B.T. , Hwang, H.S. , Kim, J.I. , Kim, D.K. and Kim, H.H. (2016) O‐glycans and O‐glycosylation sites of recombinant human GM‐CSF derived from suspension‐cultured rice cells, and their structural role. Biochem. Biophys. Res. Comm. 479, 266–271. 27638310 10.1016/j.bbrc.2016.09.057

[pbi12994-bib-0035] Kober, L. , Zehe, C. and Bode, J. (2013) Optimized signal peptides for the development of high expressing CHO cell lines. Biotechnol. Bioeng. 110, 1164–1173. 23124363 10.1002/bit.24776

[pbi12994-bib-0036] LeBowitz, J.H. , Grubb, J.H. , Maga, J.A. , Schmiel, D.H. , Vogler, C. and Sly, W.S. (2004) Glycosylation‐independent targeting enhances enzyme delivery to lysosomes and decreases storage in mucopolysaccharidosis type VII mice. Proc. Natl. Acad. Sci. USA, 101, 3083–3088. 14976248 10.1073/pnas.0308728100 PMC365748

[pbi12994-bib-0037] Lingg, N. , Zhang, P. , Song, Z. and Bardor, M. (2012) The sweet tooth of biopharmaceuticals: Importance of recombinant protein glycosylation analysis. Biotechnol. J. 7, 1462–1472. 22829536 10.1002/biot.201200078

[pbi12994-bib-0038] Liu, C. , Towler, M.J. , Medrano, G. , Cramer, C.L. and Weathers, P.J. (2009) Production of mouse interleukin‐12 is greater in tobacco hairy roots grown in a mist reactor than in an airlift reactor. Biotechnol. Bioeng. 102, 1074–1086. 18988263 10.1002/bit.22154

[pbi12994-bib-0039] Lu, J.Z. , Boado, R.J. , Hui, E.K. , Zhou, Q.H. and Pardridge, W.M. (2011) Expression in CHO cells and pharmacokinetics and brain uptake in the Rhesus monkey of an IgG‐iduronate‐2‐sulfatase fusion protein. Biotechnol. Bioeng. 108, 1954–1964. 21351076 10.1002/bit.23118 PMC3117053

[pbi12994-bib-0040] Markmann, S. , Thelen, M. , Cornils, K. , Schweizer, M. , Brocke‐Ahmadinejad, N. , Willnow, T. , Heeren, J. *et al*. (2015) Lrp1/LDL receptor play critical roles in mannose 6‐phosphate‐independent lysosomal enzyme targeting. Traffic, 16, 743–759. 25786328 10.1111/tra.12284

[pbi12994-bib-0041] Mathieu‐Rivet, E. , Scholz, M. , Arias, C. , Dardelle, F. , Schulze, S. , Le Mauff, F. , Teo, G. *et al*. (2013) Exploring the N‐glycosylation pathway in *Chlamydomonas reinhardtii* unravels novel complex structures. Mol. Cell Proteomics, 12, 3160–3183. 23912651 10.1074/mcp.M113.028191 PMC3820931

[pbi12994-bib-0042] Medina‐Bolívar, F. and Cramer, C. (2004) Production of recombinant proteins by hairy roots cultured in plastic sleeve bioreactors. In Recombinant Gene Expression: Reviews and Protocols ( Balbás, P. and Lorence, A. , eds), pp. 351–363. Totowa, NJ: Humana Press. 10.1385/1-59259-774-2:351 15269436

[pbi12994-bib-0043] Muro, S. , Schuchman, E.H. and Muzykantov, V.R. (2006) Lysosomal enzyme delivery by ICAM‐1‐targeted nanocarriers bypassing glycosylation‐ and clathrin‐dependent endocytosis. Mol. Ther. 13, 135–141. 16153895 10.1016/j.ymthe.2005.07.687

[pbi12994-bib-0044] Osborn, M.J. , McElmurry, R.T. , Peacock, B. , Tolar, J. and Blazar, B.R. (2008) Targeting of the CNS in MPS‐IH using a nonviral transferrin‐alpha‐l‐iduronidase fusion gene product. Mol. Ther. 16, 1459–1466. 10.1038/mt.2008.119 PMC2574880 18523448

[pbi12994-bib-0045] Ou, L. , Herzog, T.L. , Wilmot, C.M. and Whitley, C.B. (2014) Standardization of alpha‐L‐iduronidase enzyme assay with Michaelis‐Menten kinetics. Mol. Genet. Metab. 111, 113–115. 24332804 10.1016/j.ymgme.2013.11.009 PMC4014300

[pbi12994-bib-0046] Pham, N.B. , Schafer, H. and Wink, M. (2012) Production and secretion of recombinant thaumatin in tobacco hairy root cultures. Biotechnol. J. 7, 537–545. 22125283 10.1002/biot.201100430

[pbi12994-bib-0047] Pierce, O.M. , McNair, G.R. , He, X. , Kajiura, H. , Fujiyama, K. and Kermode, A.R. (2017) N‐glycan structures and downstream mannose‐phosphorylation of plant recombinant human alpha‐l‐iduronidase: toward development of enzyme replacement therapy for mucopolysaccharidosis I. Plant Mol. Biol. 95, 593–606. 29119347 10.1007/s11103-017-0673-x

[pbi12994-bib-0048] Pinkhasov, J. , Alvarez, M.L. , Rigano, M.M. , Piensook, K. , Larios, D. , Pabst, M. , Grass, J. *et al*. (2011) Recombinant plant‐expressed tumour‐associated MUC1 peptide is immunogenic and capable of breaking tolerance in MUC1.Tg mice. Plant Biotechnol. J. 9, 991–1001. 21740504 10.1111/j.1467-7652.2011.00614.x

[pbi12994-bib-0049] Prince, W.S. , McCormick, L.M. , Wendt, D.J. , Fitzpatrick, P.A. , Schwartz, K.L. , Aguilera, A.I. , Koppaka, V. *et al*. (2004) Lipoprotein receptor binding, cellular uptake, and lysosomal delivery of fusions between the receptor‐associated protein (RAP) and α‐l‐iduronidase or acid α‐glucosidase. J. Biol. Chem. 279, 35037–35046. 15170390 10.1074/jbc.M402630200

[pbi12994-bib-0050] Schoberer, J. and Strasser, R. (2017) Plant glyco‐biotechnology. Semin. Cell Dev. Biol. 80, 133–141. 28688929 10.1016/j.semcdb.2017.07.005

[pbi12994-bib-0051] Shaaltiel, Y. and Tekoah, Y. (2016) Plant specific N‐glycans do not have proven adverse effects in humans. Nat. Biotechnol. 34, 706–708. 27404878 10.1038/nbt.3556

[pbi12994-bib-0052] Shaaltiel, Y. , Baum, G. , Hashmueli, S. , Lewkowicz, A. and Bartfeld, D. (2015) Plant cells expressing human glucocerebrosidase. Google Patents.

[pbi12994-bib-0053] Sonoda, H. , Morimoto, H. , Yoden, E. , Koshimura, Y. , Kinoshita, M. , Golovina, G. , Takagi, H. *et al*. (2018) A blood‐brain‐barrier‐penetrating anti‐human transferrin receptor antibody fusion protein for neuronopathic mucopolysaccharidosis II. Mol. Ther. 26, 1366–1374. 29606503 10.1016/j.ymthe.2018.02.032 PMC5993955

[pbi12994-bib-0054] Srivastava, S. and Srivastava, A.K. (2007) Hairy root culture for mass‐production of high‐value secondary metabolites. Crit. Rev. Biotechnol. 27, 29–43. 17364688 10.1080/07388550601173918

[pbi12994-bib-0055] Tekoah, Y. (2006) Posttranslational modifications to plants – glycosylation. In Encyclopedia of Genetics, Genomics, Proteomics and Bioinformatics ( Jorde, L.B. , Little, P.F. , Dunn, M.J. , and Subramaniam, S. eds), 10.1002/047001153X.g305321

[pbi12994-bib-0056] Tekoah, Y. , Tzaban, S. , Kizhner, T. , Hainrichson, M. , Gantman, A. , Golembo, M. , Aviezer, D. *et al*. (2013) Glycosylation and functionality of recombinant β‐glucocerebrosidase from various production systems. Biosci. Rep. 33, e00071. 23980545 10.1042/BSR20130081 PMC3782720

[pbi12994-bib-0057] Vanier, G. , Hempel, F. , Chan, P. , Rodamer, M. , Vaudry, D. , Maier, U.G. , Lerouge, P. *et al*. (2015) Biochemical characterization of human anti‐hepatitis B monoclonal antibody produced in the microalgae *Phaeodactylum tricornutum* . PLoS ONE, 10, e0139282. 26437211 10.1371/journal.pone.0139282 PMC4593558

[pbi12994-bib-0058] Walsh, G. and Jefferis, R. (2006) Post‐translational modifications in the context of therapeutic proteins. Nat. Biotechnol. 24, 1241–1252. 17033665 10.1038/nbt1252

[pbi12994-bib-0059] Wongsamuth, R. and Doran, P.M. (1997) Production of monoclonal antibodies by tobacco hairy roots. Biotechnol. Bioeng. 54, 401–415. 18634133 10.1002/(SICI)1097-0290(19970605)54:5<401::AID-BIT1>3.0.CO;2-I

[pbi12994-bib-0060] Woods, R.R. , Geyer, B.C. and Mor, T.S. (2008) Hairy‐root organ cultures for the production of human acetylcholinesterase. BMC Biotechnol. 8, 95. 19105816 10.1186/1472-6750-8-95 PMC2648960

[pbi12994-bib-0062] Xia, H. , Mao, Q. and Davidson, B.L. (2001) The HIV Tat protein transduction domain improves the biodistribution of beta‐glucuronidase expressed from recombinant viral vectors. Nat. Biotechnol. 19, 640–644. 11433275 10.1038/90242

[pbi12994-bib-0063] Zhang, X.Y. , Dinh, A. , Cronin, J. , Li, S.C. and Reiser, J. (2008) Cellular uptake and lysosomal delivery of galactocerebrosidase tagged with the HIV Tat protein transduction domain. J. Neurochem. 104, 1055–1064. 17986221 10.1111/j.1471-4159.2007.05030.x

[pbi12994-bib-0064] Zhang, P. , Wang, T. , Bardor, M. and Song, Z. (2013) Deciphering O‐glycomics for the development and production of biopharmaceuticals. Pharm. Bioprocess. 1, 89–104.

[pbi12994-bib-0065] Zhao, K.‐W. , Faull, K.F. , Kakkis, E.D. and Neufeld, E.F. (1997) Carbohydrate structures of recombinant human α‐l‐iduronidase secreted by Chinese hamster ovary cells. J. Biol. Chem. 272, 22758–22765. 9278435 10.1074/jbc.272.36.22758

[pbi12994-bib-0066] Zimmermann, R. , Eyrisch, S. , Ahmad, M. and Helms, V. (2011) Protein translocation across the ER membrane. Biochim. Biophys. Acta, 1808, 912–924. 20599535 10.1016/j.bbamem.2010.06.015

