# Supplementary figures and images for: Brassica rapa hairy root based expression system leads to the production of highly homogenous and reproducible profiles of recombinant human alpha‐L‐iduronidase
Source: Plant Biotechnol J. 2018 Aug 30;17(2):505–16. doi: 10.1111/pbi.12994 (PMC6335068; doi:10.1111/pbi.12994)

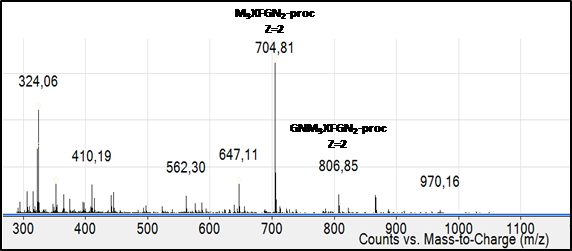

Supplement: Supplementary file 1 — Figure S1 ESI mass spectrum of N‐glycans isolated from endogenous proteins of old roots collected at day 24. [file PBI-17-505-s001.tif]
