# Supplementary material for: Brassica rapa hairy root based expression system leads to the production of highly homogenous and reproducible profiles of recombinant human alpha‐L‐iduronidase
Source: Plant Biotechnol J. 2018 Aug 30;17(2):505–16. doi: 10.1111/pbi.12994 (PMC6335068; doi:10.1111/pbi.12994)
Supplement: Supplementary file 2 — Data S1 Methodological details for the analysis of the glycosylation of endogenous protein as well as the analysis of the purity of the protein of interest. [file PBI-17-505-s002.docx]

*Brassica rapa* hairy root based expression system leads to the production of highly homogenous and reproducible profiles of recombinant human alpha-L-iduronidase.

Florian Cardon^1^, Roser Pallisse^1^, Muriel Bardor^2 ; 3^, Aurore Caron^1^, Jessica Vanier^2^, Jean Pierre Ele Ekouna^4^, Patrice Lerouge^2^, Michèle Boitel-Conti ^4^ and Marina Guillet^1^

^1^ Root Lines Technology SA, 72 Rue des Jacobins, 80000 Amiens, France

^2^ Normandie Univ, UNIROUEN, Laboratoire Glyco-MEV EA4358, 76000 Rouen, France

^3^ Institut Universitaire de France (I.U.F.) 1, rue Descartes, 75231 Paris Cedex 05, France

^4^ Biologie des plantes et innovation (BIOPI), Université de Picardie Jules Verne, 33 rue St Leu, 80039 Amiens, France

**Corresponding author**: F. Cardon, florian.cardon@rootlines-tech.com, +33(0)322092830

**Supplementary Materials and methods**

**Analysis of the glycosylation of endogenous protein**

Endogenous proteins from hairy root of *Brassica rapa* were extracted using a phosphate buffer containing PMSF protease inhibitor. The proteins were then separated on a 15 % SDS-PAGE, transferred onto a nitrocellulose membrane and blocked overnight at room temperature with 1 % of tween dissolved in TBS buffer (v/v). Affinodetection with concanavalin A (Faye and Chrispeels, 1988) and immunodetection with anti-xylose, anti-fucose and anti-Lewis^a^ antibodies (Faye et al., 1993) were carried out as described previously (Bardor et al., 2009).

After extraction, endogenous proteins from hairy root of *Brassica rapa* were digested with proteases prior to a deglycosylation using Peptide *N*-Glycosiase A (PNGase A) as previously described (Baïet et al., 2011, Mathieu-Rivet et al., 2013). Released *N*-glycans were then purified over C18 and PGC pre-packed columns (Bakker et al., 2001, Ho et al., 2012). Finally, labeling of the purified *N*-glycans to procainamide (proc) was carried out by reducing amination according to the manufacturer instructions (Ludger LTD, LudgerTag Procainamide Glycan Labeling Kit). Proc derivatized *N*-glycans were then analysed by nanoLC coupled to MS analysis using the nano-LC1200 system coupled to a QTOF 6520 mass spectrometer equipped with a nanospray source and a LC-Chip Cube interface (Agilent Technologies) as reported (Wu et al., 2017).

**RP-HPLC analysis:**

RP-HPLC was used to evaluate the purity level of IDUA protein at the completion of the purification process and also as an identity test. The purity level of IDUA protein was determined by a RP-HPLC method performed on a HPLC Agilent system (HPLC Agilent Series 1200 Infinity) equipped with a refrigerated autosampler, a quaternary pump, a column heater and a DAD detector. A volume of 100 µl of sample was injected onto a Zorbax 300 SB-C8 column (100 mm x 4.6 mm I.D., 3,5 µm particule size; Agilent) and a Zorbax 300 SB-C18 column (150 mm x 4.6 mm I.D., 5 µm particule size; Agilent), thermostatised at 60°C. The mobile phase A was water with 0.1 % (v/v) trifluoroacetic acid, and B was acetonitrile with 0.1 % trifluoroacetic acid. The samples were eluted with a linear gradient of A and B, from 0 % B (0 min) to 100 % B (10 min). The flow rate was 1 mL/min. The IDUA protein UV absorbances were recorded at 220 and 280 nm.

**Bibliography:**

Bakker, H., Bardor, M., Molthoff, J. W., Gomord, V., Elbers, I., Stevens, L. H., Jordi, W., Lommen, A., Faye, L., Lerouge, P. and Bosch, D. (2001) 'Galactose-extended glycans of antibodies produced by transgenic plants', *Proceedings of the National Academy of Sciences,* 98(5), pp. 2899-2904.

Bardor, M., Cabrera, G., Stadlmann, J., Lerouge, P., Cremata, J. A., Gomord, V. and Fitchette, A.-C. (2009) 'N-Glycosylation of Plant Recombinant Pharmaceuticals', in Faye, L. & Gomord, V. (eds.) *Recombinant Proteins From Plants: Methods and Protocols*. Totowa, NJ: Humana Press, pp. 239-264.

Baïet, B., Burel, C., Saint-Jean, B., Louvet, R., Menu-Bouaouiche, L., Kiefer-Meyer, M.-C., Mathieu-Rivet, E., Lefebvre, T., Castel, H., Carlier, A., Cadoret, J.-P., Lerouge, P. and Bardor, M. (2011) 'N-Glycans of Phaeodactylum tricornutum Diatom and Functional Characterization of Its N-Acetylglucosaminyltransferase I Enzyme', *Journal of Biological Chemistry,* 286(8), pp. 6152-6164.

Faye, L. and Chrispeels, M. J. (1988) 'Common antigenic determinants in the glycoproteins of plants, molluscs and insects', *Glycoconjugate Journal,* 5(3), pp. 245-256.

Faye, L., Gomord, V., Fitchettelaine, A. C. and Chrispeels, M. J. (1993) 'Affinity Purification of Antibodies Specific for Asn-Linked Glycans Containing α1 → 3 Fucose or β1 → 2 Xylose', *Analytical Biochemistry,* 209(1), pp. 104-108.

Ho, S. C. L., Bardor, M., Feng, H., Mariati, Tong, Y. W., Song, Z., Yap, M. G. S. and Yang, Y. (2012) 'IRES-mediated Tricistronic vectors for enhancing generation of high monoclonal antibody expressing CHO cell lines', *Journal of Biotechnology,* 157(1), pp. 130-139.

Mathieu-Rivet, E., Scholz, M., Arias, C., Dardelle, F., Schulze, S., Le Mauff, F., Teo, G., Hochmal, A. K., Blanco-Rivero, A., Loutelier-Bourhis, C., Kiefer-Meyer, M.-C., Fufezan, C., Burel, C., Lerouge, P., Martinez, F., Bardor, M. and Hippler, M. (2013) 'Exploring the N-glycosylation Pathway in Chlamydomonas reinhardtii Unravels Novel Complex Structures', *Molecular & Cellular Proteomics,* 12(11), pp. 3160-3183.

Wu, L. D., Ruhaak, L. R. and Lebrilla, C. B. (2017) 'Analysis of Milk Oligosaccharides by Mass Spectrometry', in Lauc, G. & Wuhrer, M. (eds.) *High-Throughput Glycomics and Glycoproteomics: Methods and Protocols*. New York, NY: Springer New York, pp. 121-129.
